# Deletion of the Thrombin Proteolytic Site in Neurofascin 155 Causes Disruption of Nodal and Paranodal Organization

**DOI:** 10.3389/fncel.2021.576609

**Published:** 2021-03-17

**Authors:** Dipankar J. Dutta, R. Douglas Fields

**Affiliations:** ^1^Section on Nervous System Development and Plasticity, The Eunice Kennedy Shriver National Institute of Child Health and Human Development, National Institutes of Health, Bethesda, MD, United States; ^2^The Henry M. Jackson Foundation for the Advancement of Military Medicine, Inc., Bethesda, MD, United States

**Keywords:** Neurofascin155, thrombin, fibronectinIII-like, myelin, paranode, Crispr-Cas9, septate-like junctions, demyelination

## Abstract

In the central nervous system, myelin is attached to the axon in the paranodal region by a trimolecular complex of Neurofascin155 (NF155) in the myelin membrane, interacting with Caspr1 and Contactin1 on the axolemma. Alternative splicing of a single Neurofascin transcript generates several different Neurofascins expressed by several cell types, but NF155, which is expressed by oligodendrocytes, contains a domain in the third fibronectinIII-like region of the molecule that is unique. The immunoglobulin 5–6 domain of NF155 is essential for binding to Contactin1, but less is known about the functions of the NF155-unique third fibronectinIII-like domain. Mutations and autoantibodies to this region are associated with several neurodevelopmental and demyelinating nervous system disorders. Here we used Crispr-Cas9 gene editing to delete a 9 bp sequence of NF155 in this unique domain, which has recently been identified as a thrombin binding site and implicated in plasticity of the myelin sheath. This small deletion results in dysmyelination, eversion of paranodal loops of myelin, substantial enlargement of the nodal gap, a complete loss of paranodal septate junctions, and mislocalization of Caspr1 and nodal sodium channels. The animals exhibit tremor and ataxia, and biochemical and mass spectrometric analysis indicates that while NF155 is transcribed and spliced normally, the NF155 protein is subsequently degraded, resulting in loss of the full length 155 kDa native protein. These findings reveal that this 9 bp region of NF155 in its unique third fibronectinIII-like domain is essential for stability of the protein.

## Introduction

Neurofascins are a family of cell-surface proteins of the immunoglobulin superfamily generated by alternative splicing of a single Neurofascin (NF) transcript (Hassel et al., 1997). In the CNS, oligodendroglial Neurofascin 155 (NF155) interacts with the axonal Caspr1-Contactin1 complex to form septate-like cell-adhesion junctions that attach uncompacted loops of myelin to the paranodal axon. These junctions are essential for neural impulse conduction by securing the uncompacted margin of myelin to the axon, thereby forming the paranodal loops, and maintaining separation of sodium channels in the node of Ranvier from potassium channels in the juxtaparanodal region (Banerjee et al., 2006). NF155 has a similar function in Schwann cells forming myelin on axons in the peripheral nervous system (Basak et al., 2007).

NF155 is a member of the L1-CAM family of cell adhesion molecules, with 6 immunoglobulin-like extracellular domains and four fibronectin type III-like (FNIII) extracellular regions anchored to the membrane by a short transmembrane segment (Hortsch, 1996). NF155 is produced by alternative splicing of *Neurofascin* into a NF186 form, expressed on neurons, and NF155, expressed by oligodendrocytes and localized to the paranodal region of myelinated axons (Davis et al., 1996). Binding of QKi to an RNA element (QRE2) in Nfasc intron 21 is required to promote inclusion of exons 21/22, which encodes the third FNIII-like domain that is unique to NF155 (Darbelli et al., 2016).

The biological functions of NF155 are well established and the functions of distinct domains comprising the macromolecule are being identified. Conditional deletion of NF155 in mice results in disruption of the paranodal junction and causes severe motor coordination defects and death at 16–17 days after birth (Pillai et al., 2009). Deletion of the immunoglobulin 5–6 domain in NF155, results in a truncated protein that is expressed normally. However, this domain is essential for binding of NF155 with Contactin1 and thus crucial for normal myelination. In mice with the immunoglobulin 5–6 domains deleted, the paranodal septate junctions are lost, resulting in diffusion of Caspr1 and Contactin1 from the paranodes and redistribution of the juxtaparanodal potassium channels toward the nodes (Thaxton et al., 2010).

Less is known of the functions of the unique third FNIII-like domain in NF155, but this region has been implicated in various CNS immune and neurodevelopmental disorders (Kira et al., 2018; Efthymiou et al., 2019). Homozygous mutations in the *NFASC* gene that introduces a STOP codon in AA846 (Arginine) within the unique third FNIII-like domain in NF155, results in a truncated NF155 protein, while sparing other members of the NF family including NF186. This mutation causes a severe neurodevelopmental disorder characterized by hypotonia, amimia, and areflexia (Smigiel et al., 2018).

Autoantibodies to NF155 have been detected in various central and peripheral autoimmune demyelinating pathologies including Multiple Sclerosis (MS), Guillane-Barre syndrome (GBS), Chronic Inflammatory Demyelinating Polyneuropathy (CIDP), and Combined Central and Peripheral Demyelination (CCPD) (Mathey et al., 2007; Kawamura et al., 2013; Yamasaki, 2013; Cortese et al., 2016; Burnor et al., 2018; Doppler et al., 2018). In CIDP, the NF155 autoantibodies have been traced to a domain that includes the unique third FNIII-like domain in NF155 (Ng et al., 2012). Several peripheral nerve neuropathological disorders, including GBS, CIDP, and others, are also associated with NF155 autoantibodies or genetic variants of NF155 (Ng et al., 2012).

Recent research has identified a thrombin binding site in this third FNIII-like domain of NF155 at AA924–926. Cleavage of NF155 at this site by thrombin results in detachment of paranodal loops of myelin from the axon, widening of the nodal gap, thinning of the myelin sheath, and a reduction in conduction velocity (Dutta et al., 2018). We used Crispr-Cas9 to delete the nine nucleotides corresponding to the three amino acids (AA924–926, Glycine-Arginine-Glycine) comprising the thrombin cleavage site of NF155. Surprisingly, this small deletion resulted in the complete loss of NF155 due to degradation of the proten post-synthesis, phenocopying the histological, ultrastructural, and functional outcome of complete and conditional NF155 knock outs (Sherman et al., 2005; Pillai et al., 2009). Thus this three amino acid site plays a crucial role in the stability of the NF155 protein.

## Materials and Methods

### Transgenic Mouse Production and Maintenance

We generated a transgenic mouse line with Crispr-Cas9 mediated targeted deletion of 9 base pairs in chromosome 1: 132597812–132597821 corresponding to the thrombin cleavage site spanning AA 924–926 (Glycine—Arginine—Glycine) in the native Neurofascin 155 protein. The procedure was carried out by Chengyu Liu and his team in the transgenic core facility at the National Heart Lung and Blood Institute (NHLBI), National Institutes of Health (NIH), MD, United States. P90 mice of both sexes were used for all experimental analyses.

The NF155-Tdel mutant mouse line was generated using Crispr/Cas9 following standard protocols (Wang et al., 2013). Briefly, a sgRNA (TGGTCAATGGGAGAGGTGAC) was designed to cut around AA924–926, and was synthesized using ThermoFisher’s custom *in vitro* transcription service. A single strand oligonucleotide ordered from IDT, which lacked the 9bp coding for AA924–926 (aagagacagcaggccagcttccctggtgaccgtccccggggcgtggtggcccgcctgt tc ccctacagtaactacaagctggagatggtggtggtcaat—gacgggcctcgaagtga aaccaaggaattcaccaccccagaaggag, in which the position of the deleted 9 bp is marked by a hyphen), was used as a donor for homology-mediated knockin. The sgRNA (20 ng/ul) was co-microinjected with the donor oligonucleotides (100 ng/ul) and Cas9 mRNA (50 ng/ul, ordered from Trilink BioTechnologies) into the cytoplasm of zygotes collected from C57BL/6N mice (Charles River Laboratories). Injected embryos were cultured in M16 medium (MilliporeSigma) overnight in a 37°C incubator with 6% CO_2_. In the next morning, embryos that reached the 2-cell stage of development were implanted into the oviducts of pseudopregnant surrogate mothers (CD-1 strain purchased from Charles River Laboratories). Offspring born to the foster mothers were genotyped by PCR amplification using a primer pair (forward primer: CAGTCACTACCA CCACTAACC and reverse primer: CACTTTGGTGACGGTCATTCG) followed by Sanger sequencing using forward sequencing primer (CAACCTGCCTCTGTCTCAAAGG), or amplified using a PCR primer pair (forward primer: CAACCTGCCTCTGTCTCAAAGG and reverse primer: CTGCACAGATCTCTTGATAA C) followed by Sanger sequencing using a reverse primer (CACTTTGGTGAC GGTCATTCG). Founder mice with the desired 9bp deletion were bred with wild type C57BL/6 for expanding the colonies. Results of studies on a total of 38 mice are reported.

### Genotyping and Sanger Sequencing

Genotyping of the transgenic mice was carried out by PCR amplification of tail DNA followed by Sanger Sequencing of PCR amplified DNA by Macrogen, MD, United States. The PCR primer pair sequence is as follows:

NF155TDel_F1: CAA CCT GCC TCT GTC TCA AAG G

NF155TDel_R1: CTG CAC AGA TCT CTT GAT AAG C

After the sequences were obtained, it was aligned with the wild-type sequence using Multialin webserver (Corpet, 1988) to verify deletion of the intended 9 bp region in the genome.

### Immunohistochemistry

Mice were anesthetized with Isoflurane and transcardially perfused with 4% Paraformaldehyde in 0.1M Phosphate buffer, pH 7.4. Optic nerves were collected, post-fixed in 4% Paraformaldehyde for 2 h on ice, embedded and frozen at –20°C for later longitudinal cryosectioning. Epitope retrieval was performed in Citrate buffer, pH 6.0 (Invitrogen, Camarillo, CA, United States), at 95°C for 10 min. Immunostaining with anti-pan Sodium channel (Cat#S6936, rabbit polyclonal IgG, 1:100, Sigma, Saint Louis, MO, United States) and anti-Caspr1 antibody (Cat#73-001, mouse monoclonal IgG, 1:50, NeuroMab, Davis, CA, United States) was conducted to visualize nodes of Ranvier in optic nerves. Images were captured by confocal laser microscopes (LSM510, LSM 780, LSM 710, Zeiss, Germany and Olympus Fluoview FV3000 Tokyo, Japan), using 20, 40, and 100X objective lenses and digital zoom up to 5X. Alexa fluor 488 and 594 dyes, used for immunohistochemistry in Figure 2, were excited at 500 and 590 nm respectively, and their emission were captured at 520 and 618 nm respectively. 20X imaging objective was used with a numerical aperture of 0.4 for this figure. Pinhole was set to 1 and the size of optical section was 1 micron. Results from 3 WT and 3 NF155-Tdel mice are reported.

### Electron Microscopy

Mice were anesthetized and transcardially perfused with 2.5% Glutaraldehyde and 2% Paraformaldehyde in 0.13 M Sodium Cacodylate, pH 7.4. Optic nerves were immediately removed from animals, then post-fixed in the same solution for 2 h at room temperature. Samples were post-fixed for 2 h in 2% Osmium Tetroxide. Samples were washed six times in 0.13 M Sodium Cacodylate, pH 7.4, and stained with 1% Uranyl Acetate in 0.05 M Sodium Acetate, pH 5.0, for 2 h at 4°C. Samples were then dehydrated through a graded series of ethanol solutions and infiltrated with Epon. Ultrathin sections were cut with a diamond knife, stained with Uranyl Acetate and Lead Citrate and examined by transmission electron microscopy. TEM micrographs were generated via a paid contract with electron microscopists at Science Exchange Inc. Results from 3 WT and 3 NF155-Tdel mice are reported.

### Immunoblotting

For detection of NF155, NF125, NF186, NSE, and Caspr1; freshly dissected optic nerve tissues were lysed in Tissue Protein Extraction Reagent (TPER) with protease inhibitors (Pierce, IL). The lysate was mixed with SDS-PAGE loading buffer (Thermo Fisher Scientific, MD) before electrophoresis.

**FIGURE 1:**
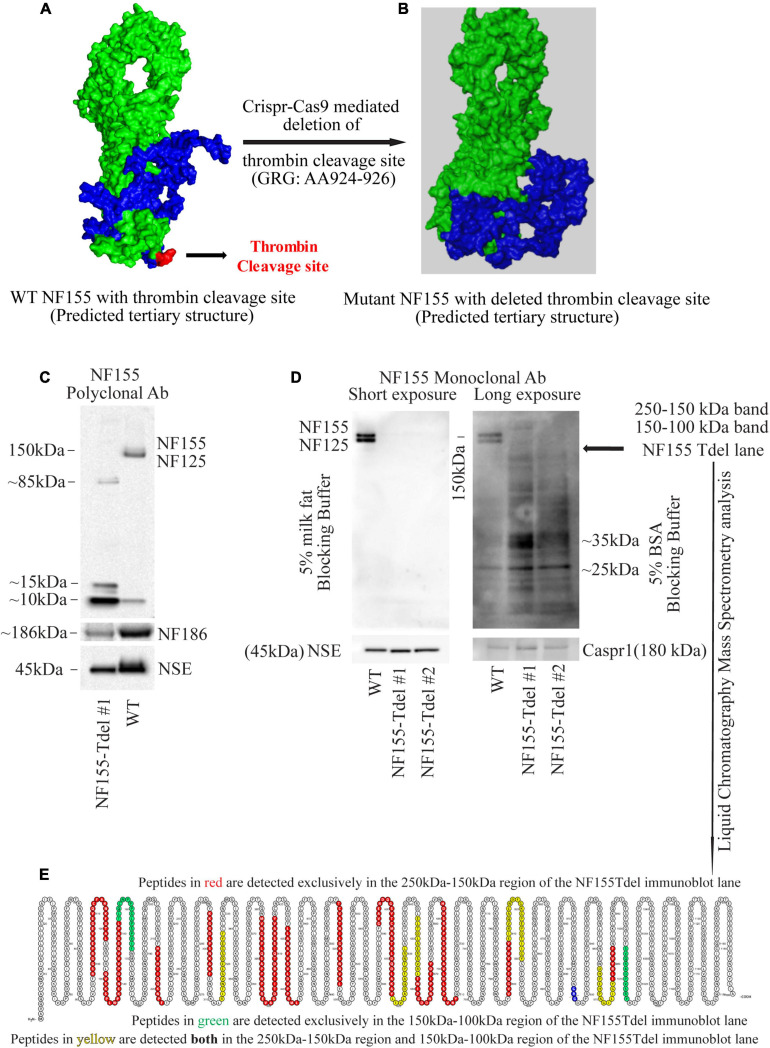
Neurofascin 155 protein is degraded upon deletion of AA924–926 (GRG) in NF155Tdel mice. The predicted tertiary structure of wild type Neurofascin 155 **(A)** is overall similar to that of Neurofascin 155 with deleted thrombin binding site spanning AA924–926 **(B)**. Green = NF125, the region of NF155 protein N-terminal to the thrombin cleavage site. Blue = NF30, the region of the NF155 protein C-terminal to the thrombin cleavage site. Red = thrombin cleavage site. A polyclonal NF155 antibody could not detect the full length NF155 (155 kDa) and its longer thrombin cleaved fragment NF125 (125 kDa) in optic nerve samples from NF155Tdel mice **(C)**. Instead, it detected smaller sized fragments weighing; 10, 15, and 85 kDa; potentially belonging to degraded NF155 fragments **(C)**. Expression of NF186 was not affected in NF155del mice **(C)**. Neuron Specific Enolase (NSE) was used as loading control, and higher levels of protein were loaded in the WT condition as a more rigorous test of possible NF155 fragments also appearing in normal conditions, but no such fragmentation was detected in WT animals **(C)**. **(D)** Use of a monoclonal antibody with 5% BSA as blocking buffer and longer exposure showed a protein smear in two independent optic nerve samples from NF155Tdel mice, and no full length NF155 or its long thrombin cleaved fragment NF125 was observed, indicating degradation of the NF155 protein. Expression and stability of Caspr1, one of the other members of the paranodal septate-like junctions, was normal in NF155Tdel mice **(D)**. Protein loading, assessed via NSE expression, was equal in all immunoblot lanes in **(D)**. Liquid Chromatography—Mass Spectroscopy analysis confirmed the presence of peptides belonging to NF155 in the 250–100 kDa region of the smeared lanes from NF155Tdel mice **(E)**.

Total protein was resolved by SDS-PAGE on 4–12% NuPAGE Bis-Tris gels (Life Technologies, Carlsbad, CA), transferred to PVDF membrane (Immobilon-P, Millipore, Bedford, MA) and blocked in Tris Buffer Solution (TBS, 10 Mm Tris-Cl, pH 7.5, 0.9% NaCl) containing 0.1% (v/v) Triton X-100 (TBST) and either 5% (w/v) non-fat milk or 5% (w/v) bovine serum albumin for 1 h at room temperature. Membranes were probed with appropriate antibodies in TBST and either 5% non-fat milk or 5% BSA overnight at 4°C. Primary antibodies were visualized with HRP-conjugated secondary antibodies (Amersham Pharmacia Biotech, Piscataway, NJ) at 1:5,000 dilution with either regular (picomolar sensitivity) or enhanced (femtomolar sensitivity) chemiluminescence (Life Technologies, Carlsbad, CA). The total protein loaded in the WT lane in Figure 1C was approximately 50% higher than that in the NF155T-del lane. Two identical trials were performed. Total protein loaded in all lanes in Figure 1D were equal. Two identical trials were performed. Results from 4 NF155-Tdel and 2 WT mice are reported. Primary antibodies used for immunoblotting: NF155 and NF125 polyclonal (Cat# ab15189, Millipore, Billerica, MA) used at 1:1,000, NF155 and NF125 monoclonal (Cat# 15035, Cell Signaling, Danvers, MA) used at 1:1,000, NF186 (Cat# ab31457, Abcam, Cambridge, MA) used at 1:1,000, NSE (Cat# ab53025, Abcam, Cambridge, MA) used at 1:2,000, and Caspr1 (Cat# ab34151, Abcam, Cambridge, MA) used at 1:1,000.

### Antibodies Used for the Detection of NF155 Cleaved Fragments

Upon cleavage of NF155 by thrombin, its two cleaved fragments were detected in immunoblots with antibodies, polyclonal and monoclonal, whose epitopes are located N-terminal to the thrombin cleavage site in NF155 at AA 924. These antibodies (Polyclonal antibody, Cat# ab15189, Millipore, Billerica, MA; Monoclonal antibody, Cat# 15035, Cell Signaling, Danvers, MA) with the N-terminal epitopes detect the full-length NF155 protein as well as its longer 125 kDa thrombin-cleaved fragment, Neurofascin 125 (NF125).

### Liquid Chromatography-Mass Spectrometry (LC-MS) Analyses

Liquid chromatography-mass spectrometry (LC-MS) was used to determine the presence of peptides corresponding to NF155 protein in immunoblots (Figure 1E) of NF155Tdel mice. Fresh optic nerve tissue lysates from NF155Tdel mice were run in a SDS-PAGE gel with size-appropriate protein ladders. Two bands on the gel corresponding to 250–150 and 150–100 kDa were excised and sent to the LC-MS facility at the National Institute of Neurological Disorders and Stroke (NINDS), NIH for protein identification analysis. Peptides identified by LC-MS as belonging to NF155 protein were rendered in a protein amino acid visualization tool, Protter (Omasits et al., 2014).

### Protein Structure Prediction

The tertiary structure of native NF155 and its various putative mutant forms resulting from mutations and deletions in its thrombin cleavage site spanning AA924–926 were predicted using I-TASSER (Iterative Threading ASSEmbly Refinement), a state-of-the-art algorithm for protein structure and function prediction (Roy et al., 2010). The predicted structures were then rendered and annotated in Polyview-3D (Porollo and Meller, 2007).

### Neurological Assessment of NF155Tdel Mice

NF155Tdel mice, beginning around 30 days after birth, exhibited sustained symptoms of ataxia which progressively got worse as the mice got older. We observed these mice regularly starting at their day of birth and until their death between 3 and 6 months of age. We observed them in their cages as well as outside their cages. The behavior of 20 mice was examined.

## Results

The neurofascin gene in mice is in chromosome 1: 132,564,690–132,741,797. All members of the neurofascin family of proteins, including NF155, are derived via alternative splicing of a single neurofascin gene transcript (Hassel et al., 1997). The thrombin cleavage site in NF155, located in the third Fibronectin-III like domain, is in a domain unique to NF155 among all other members of the Neurofascin family (Dutta et al., 2018). The 9 nucleotides corresponding to the 3 amino acid thrombin cleavage site in NF155 [Glycine (G, AA924)—Arginine (R, AA925)—Glycine (G, 926)] is located in chromosome 1: 132597812–132597821. Having determined the exact coordinates of the NF gene corresponding to the thrombin cleavage site in the NF155 protein, we proceeded to determine *in silico*, the putative effects of various mutations and deletions in this site to NF155 structure.

Predictive modeling of the NF155 protein structure suggested that this thrombin cleavage site is at the tip of a beta hairpin loop protruding away from the protein core (Figure 1A). Thrombin is capable of cleaving substrates with a wide range of amino acid sequences (Le Bonniec et al., 1996; Jenny et al., 2003). We introduced point mutations (Supplementary Figures S1A–E) and deletions (Supplementary Figures S1F–H) at and around AA924–926 region of NF155 *in silico*. Among all alternatives examined and shown in Supplementary Figures S1A–H, deletion of the three AA (GRG, AA924–926) corresponding to the entire thrombin cleavage site in NF155, yielded a predicted 3D structure that was most similar to the native NF155 tertiary structure (Figures 1A,B). Moreover, since these three AA are in a domain unique to NF155, this deletion would not have deleterious effects on other members of the Neurofascin family. We then performed Crispr-Cas9 mediated deletion of the 9 nucleotides, Chr1: 132597812–132597821, corresponding to AA924–926 in the NF155 protein, in C57Bl6 mice.

We confirmed that the mice, hereby referred to as NF155-Tdel, had the appropriate deletion by PCR amplification of the Neurofascin gene in the transgenic mice followed by Sanger sequencing of the amplified Neurofascin gene products (Supplementary Figure S1I).

Although we deleted only the three amino acids (GRG, AA924–926) corresponding to the thrombin cleavage site in the NF155-Tdel mice, we could not detect the full-length 155 kDa native protein in these mice by standard immunoblotting with an NF155 polyclonal antibody. Signs of protein degradation were evident (Figure 1C). These degraded fragments were not detected in the WT lane, even with higher protein loading in the WT lane. This implies that the bands in the NF155T-del lane in Figure 1C are indeed degraded protein fragments of NF155T-del protein, and are not non-specific protein bands. Use of a monoclonal NF155 antibody after prolonged exposure in the presence of a more sensitive chemiluminescent substrate, with and without 5% milk fat as blocking and incubation buffer, showed a protein smear where distinct protein bands should be, clearly indicating that the NF155 protein was severely degraded (Figure 1D). However, expression and stability of Caspr1 protein, one of the interacting partners of NF155 in paranodal septate-like junctions, was normal (Figure 1D). Neuron Specific Enolase (NSE) was used as protein loading controls.

We detected normal amounts and sizes of nodal Neurofascin186, the largest member of the family (Hassel et al., 1997), indicating that the alternative splicing of this neurofascin gene transcript is not affected by removal of the 9 nucleotides corresponding to the thrombin cleavage site in NF155. Since the thrombin cleavage site is located in a domain, the third Fibronectin III-like domain, which is unique to NF155, other members of the neurofascin family, as expected, were unaffected by removal of these 9 nucleotides from the neurofascin gene. NF186 was detected with a pan NF C-terminal antibody that does not detect full length NF155 as per the antibody product datasheet^1^.

Both the polyclonal and the monoclonal NF155-specific antibodies detected residues in the third FNIII domain unique to NF155. Specifically, the NF155 polyclonal antibody detects the epitope spanning AA 886–903 of the full length NF155 protein, and the NF155 monoclonal antibody detects residues surrounding AA 881 of the full length NF155 protein. Since both these antibodies recognize denatured proteins in immunoblots, there is no reason to believe that any hypothetical change in tertiary structure of NF155 in NF155Tdel mice would preclude detection of the NF155 protein in immunoblots. These data support the conclusion that the smearing and absence of full-length NF155 protein detected in NF155Tdel immunoblots is the consequence of degradation of the NF155 protein in NF155Tdel mice.

For independent confirmation of the immunoblot data, we performed liquid chromatography mass spectrometry (LC-MS) analysis of two gel regions in the NF155Tdel immunoblot lane. We excised the gel region spanning 250–150 kDa to probe for the presence of NF155 protein weighing 155 kDa. We also excised the gel region spanning 150–100 kDa to probe for the presence of NF125 protein weighing 125 kDa. The proteins in the excised gel regions were enzymatically digested, with Trypsin and Chymotrypsin, and the digested peptides were sequenced via LC-MS. Peptides from the 250 to 150 kDa region of the gel (red and yellow, Figure 1E), from full length NF155, mapped to both N-terminal and C-terminal to the thrombin cleavage site (blue, Figure 1E), as expected. However, surprisingly, 2 peptides from the 150–100 kDa region of the gel (green and yellow, Figure 1E) mapped C-terminal to the thrombin cleavage site (blue, Figure 1E). These two peptides do not belong to the NF125 protein, which is N-terminal to the thrombin cleavage site in NF155 (Dutta et al., 2018). Rather these two peptides belong to NF30, the small thrombin cleaved fragment of NF155 (Dutta et al., 2018), weighs 30 kDa, and therefore should not be present in the excised gel band spanning 150–100 kDa. In combination with the smeared NF155Tdel lanes seen in Figure 1D, this suggests that these two peptides are detected in a region they are not supposed to be detected because these peptides, in NF155Tdel mice, belong to randomly degraded fragments of NF155Tdel protein between 150 and 100 kDa in size. LC-MS detection of degraded fragments of the NF155 protein also indicates that the alternative splicing of the neurofascin transcript into NF155 transcript was unperturbed in the absence of the deleted 9 nucleotides, and that only the subsequent structural integrity of the mutated NF155 protein was affected.

Thus, the absence of the full length protein bands in sensitive immunoblots, smeared immunoblot lanes, and the presence of peptides corresponding to NF155 in the smeared immunoblot lane below 155 kDa in NF155Tdel immunoblot lanes, indicate that the NF155 protein was severely degraded in NF155Tdel mice. This indicates that the 3 AA (GRG), AA924–926, is essential for the structural integrity of the NF155 protein. In its absence, the NF155 protein is targeted for proteolytic degradation.

**FIGURE 2:**
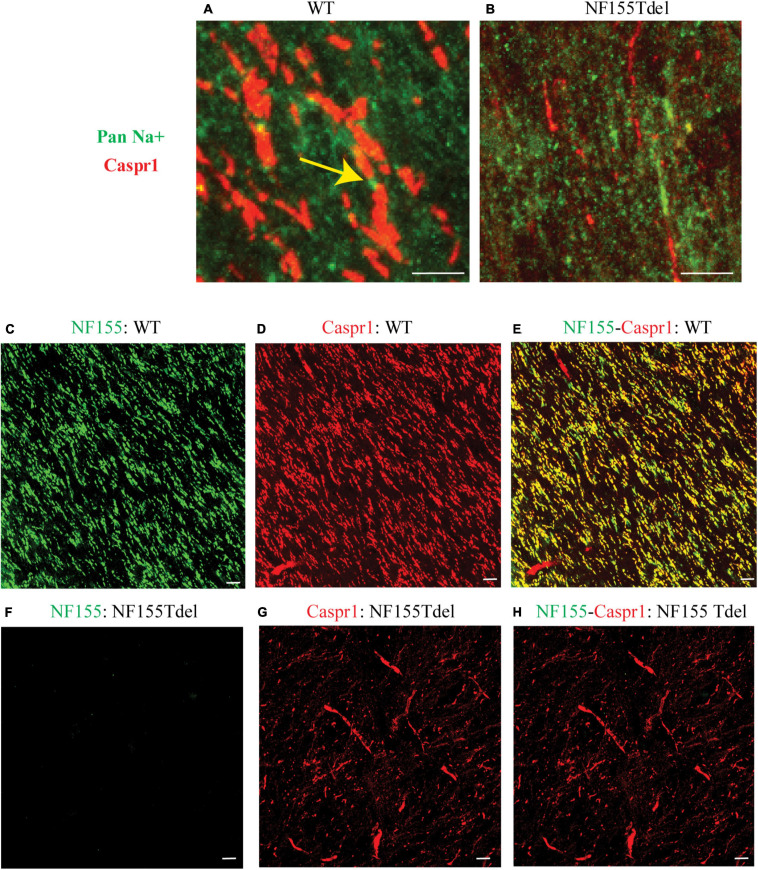
Neurofascin 155 expression is lost and Caspr1 organization is disrupted in NF155Tdel mice. Nodes of Ranvier were severely disrupted in the optic nerves of NF155 Tdel mice, and NF155 protein could not be detected. The arrangement of nodal sodium channels and paranodal Caspr1, as shown in extreme high-magnification close-ups **(A,B)** showed the normal, regular arrangement of punctate Sodium channel (green) staining at the node flanked by tapering Caspr1 (red) staining at the paranode in wild-type animals (yellow arrow in **A**), but in contrast, these features were abnormal in the NF155Tdel mice **(B)**. Wider field images at lower magnification are provided in **(C–H)**, illustrate the marked contrast between nodal structure in WT and NF155del mice. In WT mice **(C–E)**, oligodendroglial Neurofascin 155 (green, **C**) colocalizedwith its axonal interacting partner Caspr1 (red, **D**) in paranodes (yellow, **E**). However, in NF155 Tdel mice **(F–H)**, NF155 could not be detected (green, **F**) and Caspr1 staining was week and diffuse (red, **G**). Optic nerves are oriented vertically in **(A,B)** and diagonally from the upper left to lower right of the other image panels **(C–H)**. Imaging conditions were maximized to detect the faint expression of these proteins in the NF155Tdel mice **(B)** where immunostaining for these proteins was diffuse and weak as a consequence of their mislocalization in the absence of NF155. Optic nerves were imaged in WT animals using the same settings on the confocal microscope, resulting in a somewhat oversaturated image **(A)**. Using microscopy settings optimized for imaging these proteins in WT animals, staining of these proteins in the NF155Tdel mice was too dim to detect. Non-specific labeling of blood vessels in **(C–H)** could be due to non-specific interaction of the primary antibodies used with immunoglobulins inside blood vessels. Scale bar = 5μm.

To corroborate our immunoblot results of degradation of NF155 in NF155Tdel mice (Figures 1C,D), we performed immunohistochemistry of optic nerve samples from WT and NF155Tdel mice (Figure 2). WT animals exhibited normal morphology of nodes of Ranvier with sodium channels flanked by Caspr1 in paranodes of optic nerves (Figure 2A). In contrast, Caspr1 and sodium channel expression were diffuse and disorganized in NF155 Tdel mice (Figure 2B). This implies disorganization of normal paranodal structure with loss of NF155 as previously reported (Pillai et al., 2009). Confocal microscopy confirmed that oligodendroglial Neurofascin 155 (green, Figure 2C) colocalized with its interacting axonal partner Caspr1 (red, Figure 2D) in WT mice (yellow, Figure 2E). However, no Neurofascin 155 expression was detected in the optic nerve of NF155 Tdel mice (Figure 2F). Caspr1 staining was weak and not localized into paranodal domains in NF155 Tdel mice (red, Figures 2G,H). These histological results are consistent with the known functions of NF155 in forming septate junctions that attach myelin to the axon in the perinodal region.

**FIGURE 3:**
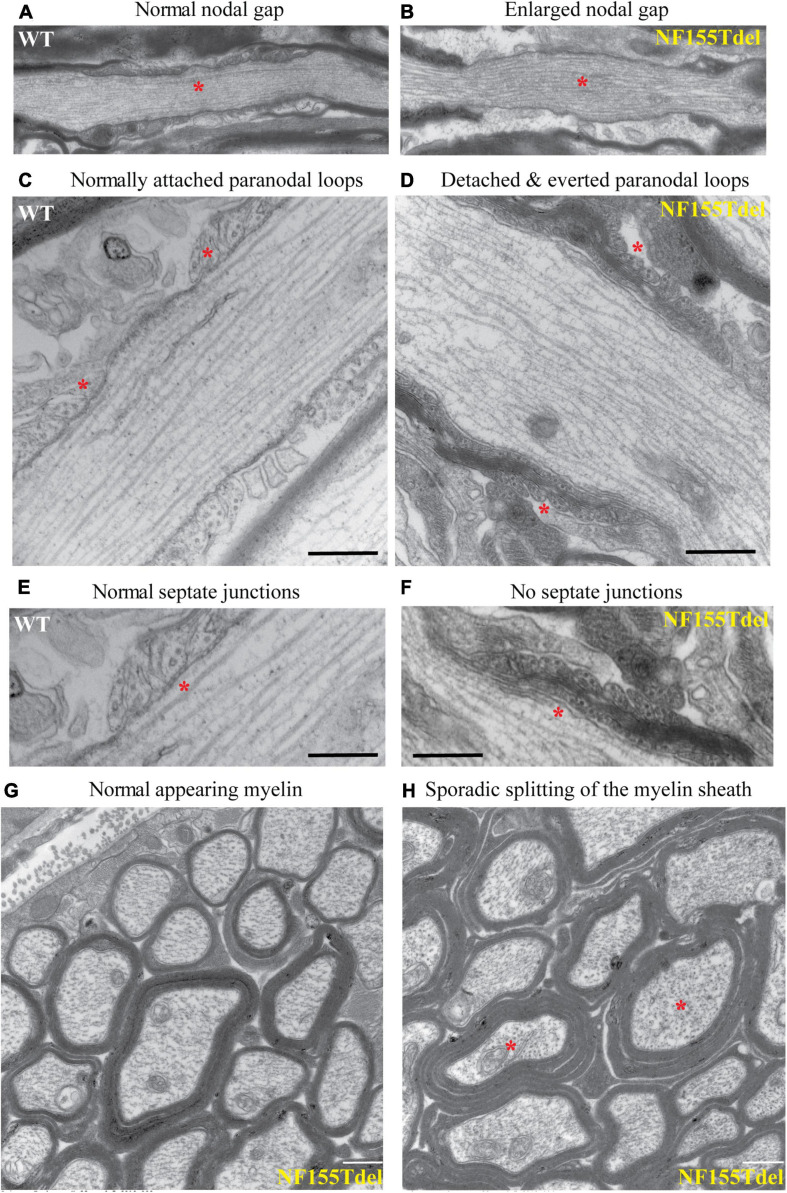
Paranodal septate-like junctions are absent, and paranodal myelin attachment to the axon is disrupted in NF155Tdel mice. Transmission electron micrographs (TEM) of wild-type optic nerve showed normal nodal gap size **(A)**, and paranodal loops attached to either side of the node normally **(C)**, via transverse electron-dense sepatate-like junctions **(E)**. In stark contrast, nodal gap was greatly expanded in NF155Tdel mice **(B)**, and paranodal loops flanking the node were detached and in most cases everted **(D)**. Sepate-like junctions were absent in the NF155 Tdel mice **(F)**. **(E,F)** Are higher magnification close-ups of **(C,D)** to show the fine structure of septate junctions and their absence in NF155Tdel mice. Although, overall myelin morphology and thickness in NF155Tdel mice appeared normal **(G)**, there was sporadic evidence of splitting of the myelin sheath in NF155Tdel mice **(H)**. * = salient events described for each panel. Scale bars = 1 μm in **(A,B)** and 0.5 μm in the rest of the images.

To examine the fine structure and predicted loss of septate junctions in NF155 Tdel mice, we performed transmission electron microscopy (TEM) of optic nerve of WT mice and NF155Tdel mice. In longitudinal sections, compared to WT mice (Figure 3A), the nodal gap was abnormally elongated in the NF155 Tdel mice (Figure 3B). In longitudinal sections, electron-dense septate-like intercellular junctions between paranodal loops of myelin and paranodal axon were present in WT mice (Figures 3C,E) and absent in NF155Tdel mice (Figures 3D,F). As expected, from the absence of septate-like junctions, the outermost paranodal myelin loops were frequently detached from the axon in NF155Tdel mice (Figure 3D) but were attached to the axon in WT mice (Figure 3C). Myelin sheath thickness appeared normal in both WT and NF155Tdel mice (Figures 3G,H). Normal thickness of the myelin sheath upon complete loss of NF155 has been described previously (Pillai et al., 2009). Myelin sheath morphology also appeared normal in NF155Tdel mice (Figures 3G,H). Normal myelin sheath morphology, upon complete loss of Nf155, has been described previously (Sherman et al., 2005; Pillai et al., 2009). However, we observed splitting of the myelin sheath in some cases in the NF155 Tdel mice (Figure 3H), giving a thicker appearance to those sheaths. Splitting of the myelin sheath in this way is one of the most common abnormalities in demyelinating conditions. With the attachments of myelin to the axon via the septate junctions missing, separation of the lamella that are no longer anchored is to be expected.

Behaviorally, the NF155Tdel mice had severe tremor and ataxia, which they started exhibiting around 1 month of age. This got worse with aging and most mice died within 3–6 months. NF155Tdel mice had abnormal gait and coordination, and exhibited sustained tremors, in comparison to their age and sex matched wild-type counterparts (Guyenet et al., 2010).

To assess their gait, we removed mice from their cages and placed them on a flat surface and observed the mice from behind while they walked. The WT mice moved normally, with body weight supported on all limbs, abdomen not touching the ground, and with both hindlimbs participating evenly. In contrast, the NF155Tdel mice exhibited tremor, appeared to limp with a lowered pelvis and feet pointing away from the body while walking (“duck feet”). These symptoms got progressively worse as the NF155Tdel mice aged and older mice had difficulty moving forward and/or dragged their abdomen on the ground while walking.

To assess their coordination, we performed the ledge test, where we lifted the mouse from their cage and placed it on the cage’s ledge. WT mice would walk along the ledge without losing balance and then lower themselves into the cage using their paws. The NF155Tdel mice, in contrast, would lose their footing while walking along the ledge and simply fall into the cage headfirst instead of landing on their paws.

To conclude, deletion of the 3 AA in NF155 protein phenocopies a complete loss of the NF155 protein. As such, the properties of the nodal gap, myelin sheath and animal behavior are similar to mice with a complete deletion of NF155. In contrast, removal of much larger domains of NF155; i.e., Imuunoglobulin 5–6 domain, has resulted in the expression of a dysfunctional but truncated NF155 protein (Pillai et al., 2009). This suggest that the 3AA, GRG (AA924–926), is required for proper folding and structural integrity of the NF155 protein.

## Discussion

Paranodal septate-like junctions are critical for proper saltatory action potential propagation in vertebrate myelinated axons (Cohen et al., 2020). Besides facilitating insulation by attaching the myelin sheath to the axon, they separate the nodal Na^+^ channels from the juxtaparanodal K^+^ channels and restrict the flow of ions between the node and the juxtaparanode (Brophy, 2001). Disruption in any of the three molecules that comprise the septate-like junctions results in similar dysmyelinating phenotypes with loss of the septate-like junctions culminating in abnormal organization of the nodal gap and the myelin sheath (Bhat et al., 2001; Boyle et al., 2001; Pillai et al., 2009). Because of the absence of a functional NF155 protein in our NF155-Tdel mice, they phenocopy mice with myelinating oligodendrocyte-specific NF155 null mutants (Charles et al., 2002; Sherman et al., 2005; Pillai et al., 2009). These mice also exhibit severe ataxia associated with everted paranodal loops, loss of paranodal axoglial junctions and failure to maintain segregated axonal domains around the node. Similar phenotype is observed in the Caspr1 KO mice and the Contactin1 KO mice (Bhat et al., 2001; Boyle et al., 2001).

The Fibronectin III domain is an evolutionarily conserved protein domain found in a wide range of extracellular proteins (Campbell and Spitzfaden, 1994). It is on average 100 amino acids long and exhibits a beta-sandwich structure. These domains are commonly located between other protein domains and function as structural spacers, to optimally arrange other domains in space (Valk et al., 2017). The thrombin cleavage site in the third FNIII-like domain is at the tip of the beta-sandwich structure, in between two opposing antiparallel beta sheets. Given the unique and essential role of FNIII domains in organizing and stabilizing globular protein structure, it is evident that even slight changes to this sequence and/or structure can be detrimental to the structure and stability of the entire protein. This is possibly the reason why larger deletions in non-fibronectin domains of NF155; for example, deletion of the entire Contactin1-interacting Immunoglobulin 5–6 domains in NF155, result in a truncated but nevertheless stable protein (Thaxton et al., 2010).

Mutations in the third Fibronectin III-like domain, a 100 amino acid stretch of the NF155 protein (aa 839–938) have been clinically associated with demyelinating neuropathies (Efthymiou et al., 2019; Monfrini et al., 2019). These disorders are similar to those associated with Neurofascin155 autoantibodies in demyelinating neuropathies (Ng et al., 2012; Doppler et al., 2018). Whether mutations in NF155 can also lead to production of NF155 autoantibodies has not been directly investigated yet, but generation of fragments of myelin proteins can be immunogenic. Our study reveals the critical importance of this three amino acid sequence (AA924–926) in NF155 protein stability, and this finding may be important for our understanding of yet to be discovered human genetic disorders resulting from targeted mutations in this critical region.

## Data Availability Statement

The original contributions presented in the study are included in the article/Supplementary Material. Further inquiries can be directed to the corresponding author/s.

## Ethics Statement

The animal study was reviewed and approved by the National Institutes of Health Animal Care and Use Committee, NICHD.

## Author Contributions

RDF and DJD conceived the project, designed experiments, analyzed data, interpreted results and prepared figures, and wrote the manuscript. DJD executed experiments and collected data. Both authors contributed to the article and approved the submitted version.

## Conflict of Interest

The authors declare that the research was conducted in the absence of any commercial or financial relationships that could be construed as a potential conflict of interest. The United States Government applied for an international patent (US Patent PCT/US2016/027776) titled, “Methods of treating or preventing demyelination using thrombin inhibitors and methods of detecting demyelination using Neurofascin 155,” on April 15, 2016, as the sole beneficiary. RF and DD are listed as inventors in the patent application.

## Footnotes

1 https://www.abcam.com/neurofascin-antibody-ab31457.html

## Supplementary Material

Supplementary Figure 1 The adverse effects of various putative mutations in the thrombin binding site of NF155 on the predicted tertiary structure of NF155. Various theoretical amino acid substitutions **(A–E)** and deletions **(F–H)** in the thrombin binding site of NF155 spanning AA924–926 adversely affect the predicted tertiary structure of NF155. The predicted tertiary structure of wild type Neurofascin 155 (Figure 1A) is most similar to that of Neurofascin 155 with deleted thrombin binding site spanning AA924–926 (Figure 1B), among all potential thrombin-site mutated NF155 structures evaluated **(A–H)**. Confirmation of Crispr-Cas9 mediated deletion of the 9 nucleotides corresponding to AA924–926 in NF155Tdel mice via Sanger sequencing of PCR amplified tail DNA.
