# Supplementary figures and images for: Deletion of the Thrombin Proteolytic Site in Neurofascin 155 Causes Disruption of Nodal and Paranodal Organization
Source: Front Cell Neurosci. 2021 Mar 17;15:576609. doi: 10.3389/fncel.2021.576609 (PMC8010152; doi:10.3389/fncel.2021.576609)

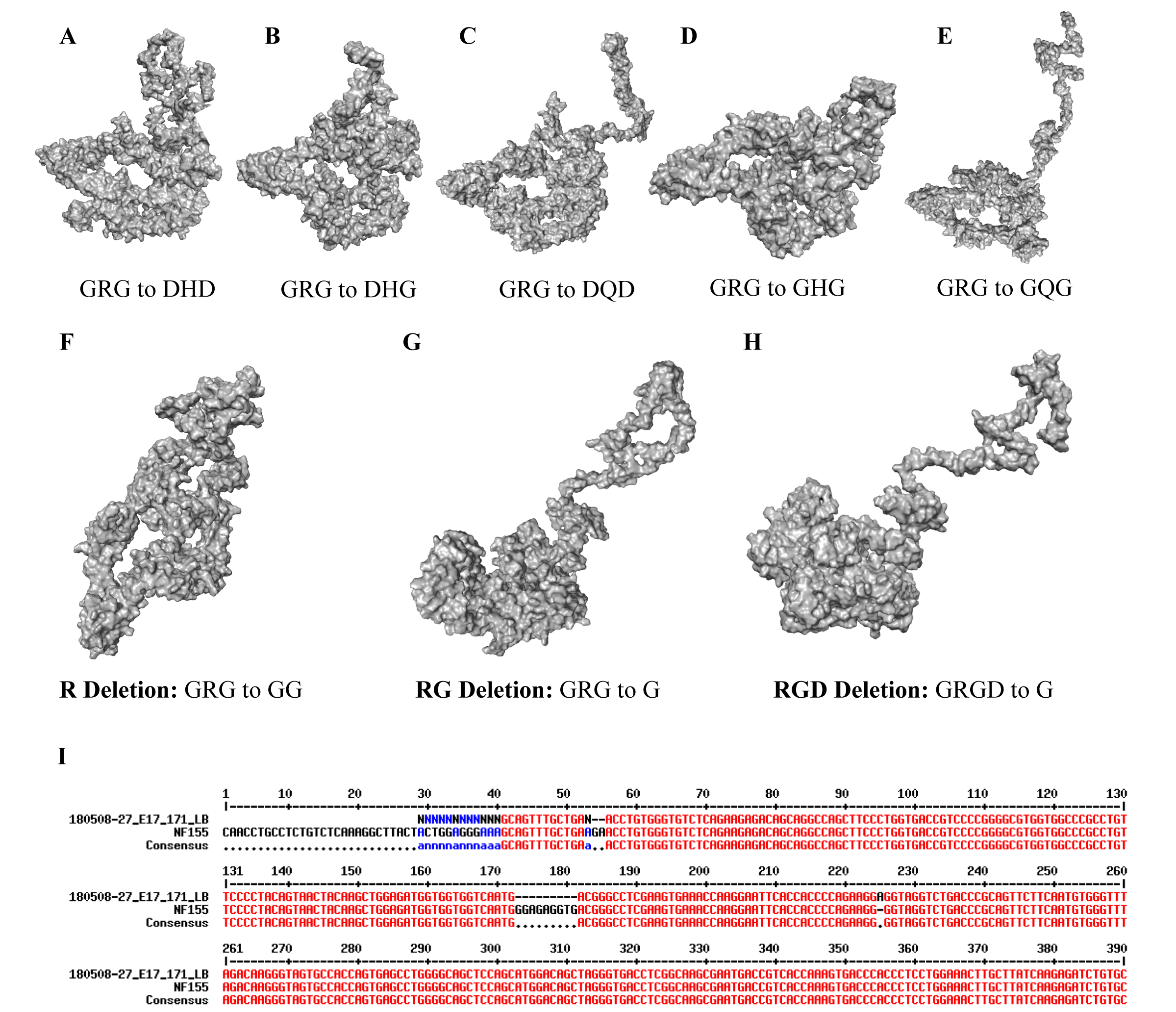

Supplement: Supplementary Figure 1 — The adverse effects of various putative mutations in the thrombin binding site of NF155 on the predicted tertiary structure of NF155. Various theoretical amino acid substitutions (A–E) and deletions (F–H) in the thrombin binding site of NF155 spanning AA924–926 adversely affect the predicted tertiary structure of NF155. The predicted tertiary structure of wild type Neurofascin 155 (Figure 1A) is most similar to that of Neurofascin 155 with deleted thrombin binding site spanning AA924–926 (Figure 1B), among all potential thrombin-site mutated NF155 structures evaluated (A–H). Confirmation of Crispr-Cas9 mediated deletion of the 9 nucleotides corresponding to AA924–926 in NF155Tdel mice via Sanger sequencing of PCR amplified tail DNA. [file Image_1.TIF]
